# Comparative performance analysis of quantum feature maps for quantum kernel-based machine learning

**DOI:** 10.1038/s41598-026-39392-9

**Published:** 2026-02-10

**Authors:** Ravi Kumar Jha, Nikola Kasabov, Saugat Bhattacharyya, Damien Coyle, Girijesh Prasad

**Affiliations:** 1 Intelligent Systems Research Centre, Ulster University, Londonderry, BT48 7JL UK; 2 Knowledge Engineering and Discovery Research Institute, Auckland University of Technology, Auckland, 1020 New Zealand; 3 Institute for Information and Communication Technologies, Bulgarian Academy of Sciences, Sofia, Bulgaria; 4 Institute for the Augmented Human, University of Bath, Bath, BA2 7AY UK

**Keywords:** Quantum kernel, Feature map, Encoding function, Classification, Machine learning, Engineering, Mathematics and computing

## Abstract

Quantum algorithms have become a popular research domain in recent times for discovering quantum-enhanced solutions in machine learning applications. Quantum kernels are one of the directions that establish such quantum-enhanced solutions to some extent. This work presents a detailed analysis of the quantum kernel approach leveraging feature maps and relevant hyperparameters to develop enhanced quantum kernels. The study includes a new high-order feature map and assesses five existing state-of-the-art feature maps for enhanced quantum kernel classifiers. Additionally, the significance of the rotational factor as a hyperparameter is highlighted for improving kernel performance. Also, it is analyzed whether different hyperparameter-tuned feature maps can lead to enhanced decision boundaries, demonstrating kernel expressivity. The analysis is undertaken on classification tasks using four different nonlinear datasets of distinct complexity. Comparative evaluations are also made with traditional machine learning models—Support Vector Machines (Linear and RBF), Naïve Bayes, Linear Discriminant Analysis, Decision Tree, Random Forest, Adaptive Boosting, and MLP. Overall, the study demonstrates that a well-tuned quantum feature map can enhance the generalization ability of quantum kernels, making them more effective for broader quantum-enhanced machine learning applications.

## Introduction

Quantum computing has gained significant popularity in the last few decades since the motivation of a quantum computer was highlighted by Richard Feynman in 1982^1^, and since then the quest for an ideal quantum computer has been an ongoing endeavour^2^. Quantum computing uses quantum information processing inspired by quantum mechanical properties, such as superposition, entanglement, and interference^3^. These quantum principles are distinct from the classical information processing and are the building blocks of quantum advantages over classical computers. For instance, the superposition of quantum bits (qubits) allows simultaneous quantum information processing, providing parallelism^4^, and the entanglement process helps achieve speed-up^5^. These properties help quantum algorithms to achieve significant advantages as reported in previous studies^4–6^. As a promising field, various notable quantum algorithms and technological advancements have been discovered and presented^4,5,7,8^.

In recent times, quantum algorithms for machine learning (ML) tasks have been evolving, and many promising algorithms have been developed^9–11^, leading to numerous quantum machine learning (QML) applications^12,13^. The motivation of QML is derived from its ability to solve complex problems efficiently, leveraging potential quantum advantages in domains like optimization, healthcare, chemistry, finance, computer vision, and many more^11,14–16^. QMLs are increasingly seen as promising candidates for leveraging the scalability of quantum computers to achieve computing advantages such as better solutions and speed-up^15–17^. Havlíček et al.^9^ proposed the use of a quantum kernel estimator in a supervised learning framework that demonstrated quantum advantage by achieving higher accuracy over synthetic datasets. Subsequent studies have focused on exploring and identifying quantum-enhanced algorithms to address a wide range of complexities in real-world datasets^18–25^. Real-life data presents various complexities, such as high-order nonlinearity in the original space and multi-dimensional features, which pose challenges for general ML algorithms.

Quantum algorithms are rapidly advancing from theoretical concepts to experimental applications in the noisy intermediate-scale quantum (NISQ) era^26^. However, developing a robust quantum model that can outperform the classical model remains challenging, and thus prompts ongoing research in quantum algorithm design, error correction, and hardware scalability^27,28^. Many quantum algorithms require more hardware resources than currently available in NISQ computers, limiting the range and scale of problems they can efficiently solve. Consequently, more research is focused on hybrid classical-quantum approaches that leverage the strengths of both classical and quantum resources to achieve computational advantages^29–32^. Hybrid classical-quantum models have proven valuable in leveraging the strengths of both paradigms. Therefore, hybrid approaches could be beneficial in managing model parameters and cost overheads^11,15,16,32^. Furthermore, a hybrid model minimizes quantum resources and improves scalability with certain challenges such as quantum state preparations and gate errors^24,25^.

Various studies have found the quantum kernel framework useful for tasks such as classification, regression, and solving complex equations^23,33–36^. In forming a quantum kernel, a feature map plays an important role. A feature map consists of an encoding function to transform the real-valued inputs into quantum state preparations in the feature space. Suzuki et al.^37^ proposed five different feature maps and their role in estimating data patterns leading to classification advantages using quantum kernels. Subsequent works have highlighted case studies in developing useful applications with real-world datasets^21–23^. However, it is not trivial to identify the most appropriate feature map. Additionally, it is worth highlighting the other significant entities responsible for developing an improved quantum kernel known as *hyperparameters*. To tap the maximum potential of a quantum kernel, it is necessary to engage with all the entities in a quantum kernel formulation. This study thus aims to highlight the importance of all entities using four different 2D benchmark datasets. To this end, novel contributions of this study are as follows:

- A new feature map has been proposed to overcome the limitation of one of the state-of-the-art feature maps developed by Suzuki et al.^37^;
- Comprehensive analyzes have been undertaken to identify the importance of the hyperparameter, useful for the performance enhancement of quantum kernels.

The remainder of the paper is organized as follows: the Methods section introduces the quantum kernel framework and outlines implementation details, including the feature maps used, datasets, and evaluation criteria. This is followed by the Results and Discussion, and finally, the Conclusion.

## Quantum kernel

In general, a kernel method projects the data from a lower-dimension into a higher-dimension space to improve the data separability, making the classification easier either through a linear or a nonlinear transformation. A nonlinear kernel is likely to be more useful over complex data and one of the popular nonlinear kernels is the radial basis function (RBF). Mathematically, a kernel represents the similarity between a data pair in the feature space and is written as: $K(x,x^{\prime}) = \langle \phi (x), \phi (x^{\prime}) \rangle$, where $\phi (x)$ infers a feature map^38^. Principally, a classical kernel can be adapted to a quantum kernel, where a quantum state space is governed by the measurement process^2,39^. A quantum kernel can be estimated by a quantum feature map circuit and integrated into a support vector machine (SVM) framework to create a classifier called a quantum kernel or quantum support vector classifier^40–42^.

**Fig. 1:**
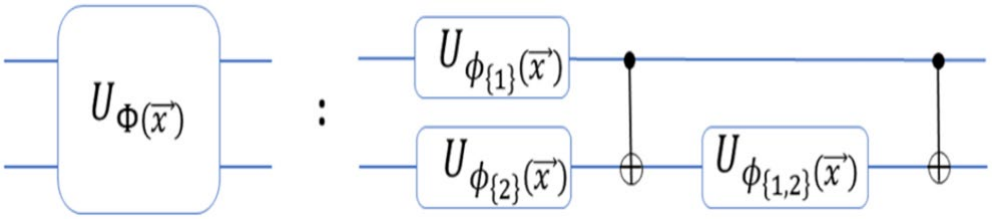
A quantum feature map circuit expanded up to the second order using encoding functions $\phi _{\{i\}}$, unitary (U), and CNOT gates^37^.

Quantum feature maps are crucial in mapping classical inputs into quantum feature space. Ideally, a better mapping function should be expressive enough to predict the underlying data patterns while providing variable separability^40,41,43^. An initial two-qubit experiment using a quantum feature map defined as a ZZ feature map provided improved classification performances for synthetic datasets^9,41^. A two-qubit feature map circuit with an encoding function $(\phi )$ and gates up to second order can be visualized in Fig. 1. A two-layer quantum circuit can make the resulting quantum kernel sufficiently complex to be classically intractable. This computational complexity justifies repeating the circuit twice to enhance the effectiveness of the quantum circuit^9,22,44^.

Here, a feature map of $n-qubits$ for real-valued input $\vec {x} \in \mathbb {R}^n$ is defined as:

1 $$\begin{aligned} U_{\Phi (\vec {x})} = \exp \left( i \sum _{j=1}^{n} \alpha _j \phi _S(\vec {x}) \prod P_j \right) \end{aligned}$$

where $P_j \in \{ I, X, Y, Z \}$ are the single qubit Pauli gates, and $\phi _{S} (\vec {x})$ represents the nonlinear data encoding functions of order $S$ expansions, where $|S| \le 2$^9,17^. The variable $\alpha$ acts as the rotational factor that complements the phase rotation of a qubit while encoding the real value input in the quantum feature space. The variable such as Pauli gates and rotational factor play significant role in preparing a feature map and can be regarded as *hyperparameters*^45^. A two-qubit ($n=2$) feature map can be composed of multiple gates and is defined by using a unitary operator $U_{\Phi (x)}$ with the Hadamard gates as:

$$\mathscr {U}_{\Phi (\vec {x})} = U_{\Phi (\vec {x})} \, H^{\otimes 2} \, U_{\Phi (\vec {x})} \, H^{\otimes 2}.$$

Here, $\Phi (\vec {x})$ is the function used to transform the real value input a higher-dimensional *Hilbert* space and given by:

$$\Phi (\vec {x}) = \{ \phi _1(x), \phi _2(x), \phi _{1,2}(x) \}$$

where $\Phi (\vec {x})$ represents a high-order nonlinear encoding function, with a circuit initializing at states $|0\rangle$^9,37^.

A quantum feature map circuit prepares data-dependent quantum states for two data points $\vec {x}_i$ and $\vec {x}_j$, which are used to estimate the entries of the kernel matrix. The circuit consists of an initial layer of Hadamard gates, followed by data-dependent single-qubit rotations and a two-qubit entangling operation implemented using a CNOT gate. The entangling pattern is linear between the two qubits. The feature-map block is repeated twice. The quantum kernel matrix, $K(\vec {x}_i, \vec {x}_j)$, is computed using a fidelity-based approach by evaluating the overlap between the corresponding quantum states of $\vec {x}_i$ and $\vec {x}_j$, and the same is defined as^9,37,40^:

2 $$\begin{aligned} K(\vec {x}_i, \vec {x}_j) = \left| \langle 0| \mathscr {U}_{\Phi (\vec {x}_j)}^{\dagger } \mathscr {U}_{\Phi (\vec {x}_i)} |0\rangle \right| ^2. \end{aligned}$$

**Fig. 2:**
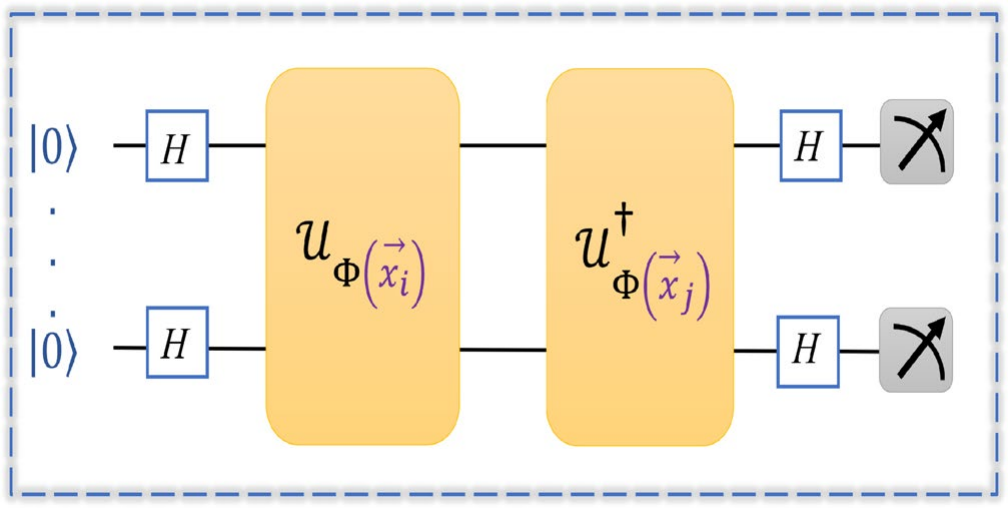
A circuit designed for quantum kernel estimate induced by feature maps initialized with $|0\rangle$ states and followed by Hadamard gates for the superposition.

The resulting kernel matrix (*K*) is then incorporated into a standard SVM and used as a quantum kernel. SVM training is performed using the standard scikit-learn *SVC* solver with a precomputed kernel (*K*). Figure 2 provides a quantum circuit design for the quantum kernel implementation on a noiseless quantum simulator. The kernel circuit structure remains the same across datasets, while the feature-map encoding functions vary. This variational quantum circuit-based approach facilitates the construction of a separating hyperplane in the quantum feature space, while simultaneously enabling the estimation of the quantum kernel on a quantum processor^45,46^. The quantum kernel formulation is summarized in Algorithm 1 and provided in the Supplementary Material (Appendix 1).

The exact classical evaluation of the inner product (i.e., kernel) between two quantum states produced by a circuit $\mathscr {U}_{\Phi (x)}$ is known to be #P-hard, as it corresponds to computing a Tutte partition function, a task that is computationally hard to simulate classically^43,47^. Achieving significant advantage in the current quantum landscape is non-trivial and challenging, particularly with classical datasets inspired by real-life applications. However, recent promising studies have shown enhanced performance by transitioning from experimental studies and synthetic datasets to real-life applications^9,21,23,37,40,41,43,48^. Building on this progress, the proposed study aims to highlight advantages of quantum-enhanced kernels induced by feature maps for ML tasks.

## Implementation details

### Feature maps

A quantum feature map comprises of nonlinear encoding functions that are essential for generating an effective kernel estimate in the quantum space. The standard procedure for developing a quantum kernel estimate for classical input is to define a feature map that translates an input vector from classical feature $\Phi (\vec {x})$ to quantum feature $|\Phi (\vec {x}) \rangle$state. It encodes input to quantum feature space with the help of single and two qubit unitary operators^39,42^. The approach for formulating a feature map can be regarded as heuristic, non-trivial, and requires consideration of multiple candidate maps^21,37^. However, the discriminating capability of a feature map can be visualized a priori through repeated measurements of quantum state spaces. Some early known feature maps are Z-Feature Map of single order, and ZZ-Feature Map up to second order expansions in the quantum space^9,21^. In this study, we use the Pauli-Feature Map up to second order expansion with different encoding functions, and named them as different feature maps throughout.

Real-world datasets are generally nonlinear and multi-dimensional. Previous studies have suggested that tuning nonlinear encoding functions for multi-dimensional complex datasets can improve classification results^21,37^. Furthermore, quantum feature maps have demonstrated their ability to capture complex data patterns effectively than traditional ML methods, thereby highlighting the advantages of quantum classification models^37,41^. Building on these insights, this work introduces a novel feature map (F1) that leverages a high-order nonlinear encoding function (Eq. 3). In addition, we analyzed other state-of-the-art feature maps (F2–F6) (Eq. 4–8) proposed by Suzuki et al.^37^ and conducted comparative evaluations as part of the proof-of-concept approach in detail. A complete quantum feature map circuit design, highlighting the number of qubits, layers, and entanglement sequence for quantum kernel estimation, is provided in the Supplementary Material (Appendix 2).

3 $$\begin{aligned} F1&: \quad \phi _{\{i\}}(x) = x_i, \quad \text {and} \quad \phi _{\{1,2\}}(x) = \frac{\pi }{{(1 + \cos (x_1))(1 + \cos (x_2))}} \end{aligned}$$

4 $$\begin{aligned} F2&: \quad \phi _{\{i\}}(x) = x_i, \quad \text {and} \quad \phi _{\{1,2\}}(x) = \pi x_1 x_2 \end{aligned}$$

5 $$\begin{aligned} F3&: \quad \phi _{\{i\}}(x) = x_i, \quad \text {and} \quad \phi _{\{1,2\}}(x) = \frac{\pi }{2}(1 - x_1)(1 - x_2) \end{aligned}$$

6 $$\begin{aligned} F4&: \quad \phi _{\{i\}}(x) = x_i, \quad \text {and} \quad \phi _{\{1,2\}}(x) = \exp \left( \frac{|x_1 - x_2|^2}{\frac{8}{\ln (\pi )}}\right) \end{aligned}$$

7 $$\begin{aligned} F5&: \quad \phi _{\{i\}}(x) = x_i, \quad \text {and} \quad \phi _{\{1,2\}}(x) = \frac{\pi }{3 \cos (x_1) \cos (x_2)} \end{aligned}$$

8 $$\begin{aligned} F6&: \quad \phi _{\{i\}}(x) = x_i, \quad \text {and} \quad \phi _{\{1,2\}}(x) = \pi \cos (x_1) \cos (x_2) \end{aligned}$$

Mathematically, the proposed encoding function F1 addresses challenges posed by other encoding functions, such as the sensitivity issues associated with encoding function F5, where *cos*(*x*) approaches zero. In such cases, F1 mitigates the sensitivity of F5 effectively. Notably, a feature map substantially impacts the generalization capability of a quantum kernel, highlighting its critical role in determining the performance of a feature map. Furthermore, hyperparameters are critical factors that influence the performance of quantum models. The rotational factor $\alpha$ controls the scale of the data encoding process and is therefore treated as a hyperparameter for the quantum feature map. Proper tuning of these hyperparameters in a feature map can significantly enhance the quantum kernel performance in ML applications. Our study delves deeper into the role and analysis of hyperparameters in upcoming sections.

### Dataset

**Fig. 3:**
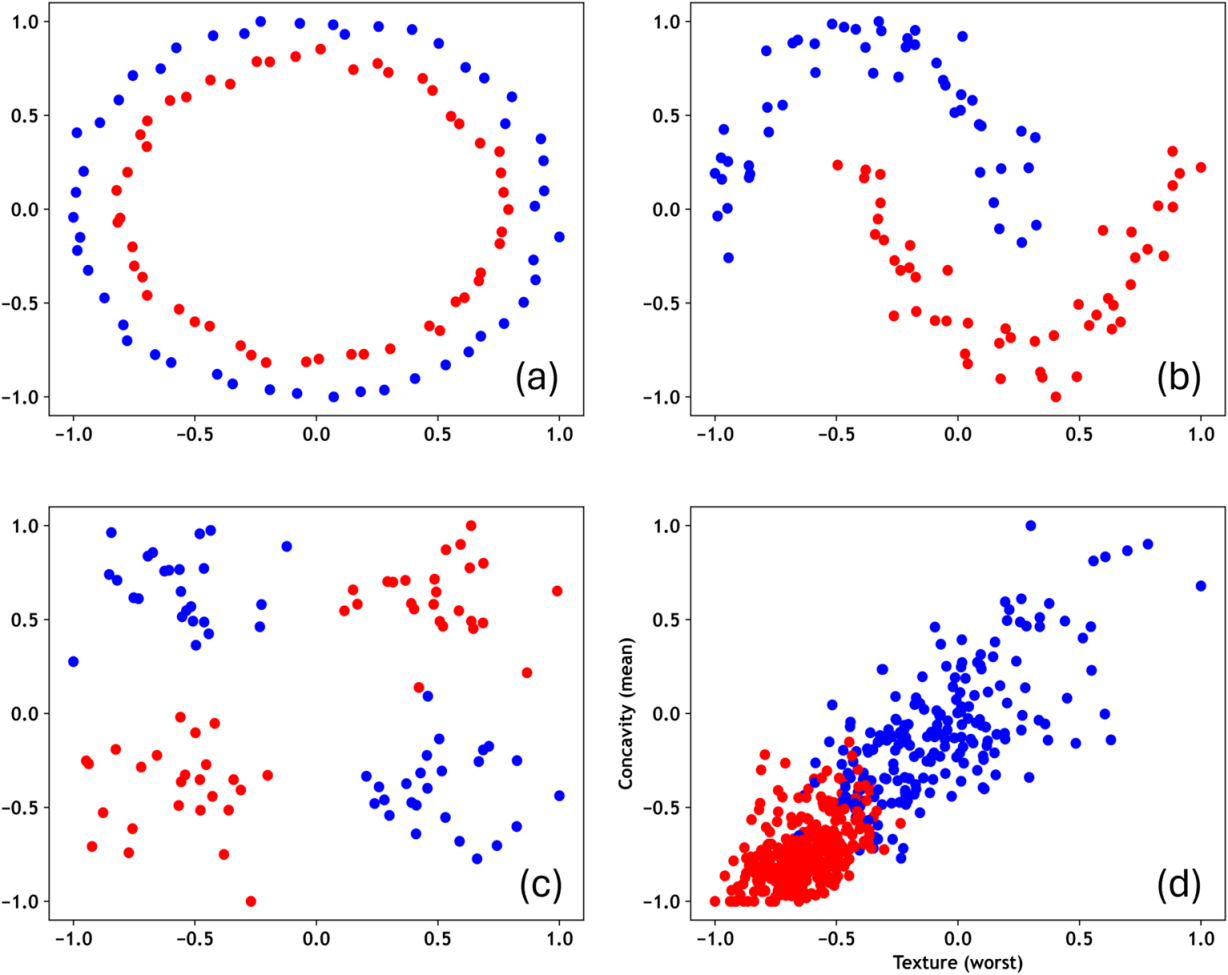
Four different benchmark nonlinear datasets: (**a**) Circle, (**b**) Moon, (**c**) XOR, and (**d**) the Wisconsin Breast Cancer (WBC) Diagnostic are used for generation and evaluation of enhanced quantum kernels.

Here we have analyzed a set of nonlinear 2D datasets namely Circle, Moon, and XOR for the binary classification tasks. Circle and Moon are imported through the *scikit-learn* library, with noise levels of 0.025 and 0.10, respectively. XOR is generated by sampling four Gaussian clusters with standard deviation 0.25, centered within the interval [− 1, +1]. Each datasets are balanced and consists of $N=100$ samples $(x_i, y_i)$ and categorized into two labels $y_i \in \{-1, +1\}$, as shown in Fig. 3a–c. Furthermore, the approach is extended to include the Wisconsin Breast Cancer (WBC) Diagnostic dataset in Fig. 3d, which introduces higher data complexity to the classification methodology while also expanding its applicability to real-world scenarios. Unlike synthetic datasets such as Circle, Moon, and XOR, WBC does not follow a known data distribution. Therefore, its inclusion serves as an extended case study to evaluate the effectiveness of enhanced quantum kernels. WBC features are derived from digitized images of breast cancer samples obtained via fine-needle aspirate. The dataset includes 569 instances with 30 features and is divided into two classes: Benign (357 instances) and Malignant (212 instances)^49^. As the experiment is designed for two-qubit simulation, we have selected the two best features, texture (worst) and concavity (mean), using a statistical approach called Boruta algorithm^50^. Boruta algorithm is an extension of a Random Forest method for identifying the most meaningful features by eliminating less significant features iteratively^50^. Finally, the input features are scaled to the interval [− 1, +1], following the implementation of Suzuki et al.^37^, prior to data encoding using quantum feature maps.

The dimension of the quantum *Hilbert* space for the data encoding using feature maps grows exponentially with the number of qubits (*n*), and features (*m*). A feature map uses exactly the same number of qubits as the number of features, i.e., $n=m$. Additionally, performing large-scale quantum simulations with an increasing number of qubits demands substantial computational resources on modern classical hardware, posing significant challenges. Therefore, in the context of hybrid models, we conduct benchmarking experiments using a shallow two-qubit quantum circuit, sufficient to emphasize the importance of feature maps and hyperparameter tuning.

### Evaluation criteria

Enhanced quantum kernels for classification tasks have been evaluated across a diverse spectrum of classical models and performance metrics. To do so, we have assessed linear and RBF kernels, Naïve Bayes (NB) probabilistic model, Linear Discriminant Analysis (LDA) model, Decision Tree (DT) nonlinear model, Random Forest (RF) ensemble model, Adaptive Boosting (AB) model, and an MLP classifier^51^. The outcomes are evaluated using different statistical metrics such as classification accuracy, standard deviation, and Matthews correlation coefficient (MCC) using the mean value of the 5-fold cross-validation on test datasets^52^. For 5-fold cross-validation, the dataset is equally partitioned into five subsets. In each fold, one subset is used for testing while the remaining subsets serve as training data. To ensure the model’s robustness, the data within each fold is shuffled and randomized. Default SVM hyperparameters are used uniformly across all quantum kernel experiments, and no additional SVM hyperparameters are tuned. Therefore, the emphasis of this work is on assessing the performance of the quantum kernels through hyperparameter-tuned quantum feature maps. In contrast, classical baseline models are optimized using grid search to ensure a rigorous comparison.

The MCC is used for its reliability as a statistical metric, offering a high score only when strong performance across all four categories of the confusion matrix (TP, FN, TN, FP) is predicted. Additionally, MCC accounts for the balance between positive and negative elements in the data, and is expressed as follows:

$$\text {MCC} = \frac{(\text {TP} \cdot \text {TN}) - (\text {FP} \cdot \text {FN})}{\sqrt{(\text {TP} + \text {FP}) \cdot (\text {TP} + \text {FN}) \cdot (\text {TN} + \text {FP}) \cdot (\text {TN} + \text {FN})}}.$$

MCC ranges from − 1 to +1, with − 1 indicating complete misclassification, +1 representing perfect classification, and 0 corresponding to the performance of a random classifier. In the results, the MCC values are maintained within the original range . It also serves as a reliable metric for binary classification tasks, providing a more comprehensive evaluation than metrics like F1 score and accuracy^52^. MCC is a suitable metric for the binary classification task, as it robustly accounts for class imbalance on WBC data. Consequently, MCC ensures a reliable evaluation without stratified folds for WBC, allowing for a consistent experimental design across all benchmark datasets^52^. The results are implemented in the PennyLane open-source Python package using *lightning.qubit* simulator on a classical computer^53^.

## Results and discussion

Here, we demonstrate and analyze enhanced quantum kernel results induced by different feature maps for classification tasks following the implementation details described above. The study evaluates the performance of the proposed feature map (F1) against five other state-of-the-art feature maps (F2–F6) and several classical baseline models. The quantum feature maps are formulated using fixed values of the rotational factor $\alpha$, specified as part of the feature-map design, and their performances are evaluated. Crucially, no additional SVM hyperparameters are tuned for the feature map–induced quantum kernels. This setup allows to isolate the effect of the rotational factor and feature map on classification performance, and to analyze the resulting kernel expressivity. In contrast, classical baseline models are tuned using a cross-validated grid search to ensure a competitive baseline, and the optimal values are provided in the Supplementary Material (Appendix 3). Table 1 provides the best performances of both quantum and classical models.

**Table 1:** Classification results are reported using test accuracy (± standard deviation) and MCC scores.

Data	Circle		Moon		XOR		WBC	
Metrics	Accuracy	MCC	Accuracy	MCC	Accuracy	MCC	Accuracy	MCC
F1	**100.0 ± 0.0**	**1.000**	97.0 ± 2.45	0.937	**99.0 ± 2.0**	**0.979**	**93.67 ± 1.40**	**0.866**
F2	90.0 ± 5.48	0.821	70.0 ± 5.48	0.487	99.0 ± 2.0	0.979	93.10 ± 1.83	0.833
F3	74.0 ± 5.83	0.499	94.0 ± 3.74	0.869	99.0 ± 2.0	0.979	92.79 ± 1.16	0.849
F4	**100.0 ± 0.0**	**1.000**	**98.0 ± 2.45**	**0.960**	99.0 ± 2.0	0.979	**93.67 ± 1.40**	**0.866**
F5	**100.0 ± 0.0**	**1.000**	97.0 ± 2.45	0.936	99.0 ± 2.0	0.979	92.09 ± 0.56	0.831
F6	**100.0 ± 0.0**	**1.000**	88.0 ± 5.10	0.754	99.0 ± 2.0	0.979	93.32 ± 1.52	0.860
LIN	43.0 ± 2.44	− 0.646	90.0 ± 5.47	0.798	52.0 ± 10.77	0.218	93.32 ± 1.61	0.859
RBF	100.0 ± 0.0	1.000	98.0 ± 2.44	0.961	99.0 ± 2.0	0.979	93.49 ± 1.62	0.862
NB	57.0 ± 10.77	0.179	89.0 ± 7.34	0.778	35.0 ± 15.81	− 0.239	92.96 ± 1.67	0.852
LDA	36.0 ± 6.63	− 0.262	89.0 ± 7.34	0.778	38.0 ± 17.20	− 0.179	91.74 ± 2.03	0.827
DT	82.0 ± 6.78	0.650	93.0 ± 4.0	0.856	92.0 ± 6.78	0.856	91.91 ± 0.65	0.828
RF	82.0 ± 5.09	0.648	97.0 ± 4.0	0.940	96.0 ± 2.0	0.920	93.15 ± 1.49	0.853
AB	92.0 ± 6.78	0.836	97.0 ± 2.44	0.942	98.0 ± 2.44	0.960	92.09 ± 1.10	0.832
MLP	51.0 ± 6.63	0.235	87.0 ± 4.0	0.746	86.0 ± 5.83	0.749	93.50 ± 1.42	0.862

The table presents the best outcomes of enhanced quantum kernels and optimized classical models.

Best values are in bold.

For Circle dataset, the circuit consists of single-qubit X-rotations followed by a data-dependent ZZ entangling interaction. Circle results show the advantages of using enhanced quantum kernels over most of the classical models. The results demonstrate favourable performance when utilizing quantum feature maps, particularly with feature maps F1 and F4–F6, as supported by the accuracy of 100.0 ± 0.0%, and perfect MCC scores of 1.000. Notably, the encoding functions of feature maps F2 and F3 exhibit a lower degree of complexity, which could be one of the reasons for their inability to match the performance of the other feature maps. Among the classical models, RBF kernel demonstrated the highest performance, followed by AB. In contrast, models like LIN kernel and LDA underperformed, likely due to the complex data patterns and inherent limitations such as less effective decision boundaries.

For Moon dataset, the circuit consists of single-qubit Y-rotations followed by a data-dependent ZZ entangling interaction. Moon results also show the advantages of using enhanced quantum kernels over most classical models, with certain variations compared to Circle. Feature maps F1, F4, and F5 are the top-performing kernels in quantum models with a minimum accuracy and MCC score of 97.0 ± 2.45% and 0.936 respectively. In particular, F4 turned out as the best-performing quantum model with an accuracy of 98.0 ± 2.45%, supported by MCC score of 0.960. F2 and F6 maps turned out as the two least performing quantum kernels, where F2 came out as the least performing quantum model with an accuracy of 70.0 ± 5.48%, supported by MCC score of 0.487. In classical models, again RBF kernel exhibited the best performance, followed by AB, and RF models. Also, MLP, NB, LDA, and LIN kernel are least performers in the given order.

For XOR dataset, the circuit consists of single-qubit X-rotations followed by a data-dependent ZZ entangling interaction. The results for XOR show that quantum kernels are beneficial with similar evaluation metrics with accuracy and MCC scores of 99.0 ± 2.0% and 0.979, respectively. Among the classical models, RBF turned out as the best performer with accuracy and MCC similar to quantum models, followed by AB (98.0 ± 2.44%) and RF(96.0 ± 2.0%). NB, LDA, LIN, and MLP are the least performing models in the given order. Further discussion is provided in the next section.

For WBC dataset, the circuit consists of single-qubit Z-rotations followed by a data-dependent ZZ entangling interaction. The results for WBC data showed competitive outcomes both within and between the quantum and classical models relative to the datasets analyzed earlier. Accuracy (93.67 ± 1.40%) and MCC score (0.866) support F1 and F4 as the two best performing models within quantum kernels, whereas F2 with MCC value of 0.833 and F5 with MCC value of 0.831 are two underperformed quantum models. MCC offers a more balanced assessment of model performance for WBC, considering the class imbalance in WBC data. Among the classical models, MLP (MCC of 0.862) and RBF (MCC of 0.862) have demonstrated better performances closer to the best-performing quantum kernels. Both LIN kernel and RF model have provided better results compared to their performances on previously analyzed datasets, demonstrating the enhanced quantum classifier’s variability across different data patterns. Also, LDA and DT are the two leastperforming models. Overall, the enhanced quantum kernels demonstrated advantages in classification tasks across different data patterns. The results highlight that the proposed quantum kernel design enhances kernel performance by amplifying the role of feature maps in effectively approximating data patterns.

### Hyperparameter analysis

In quantum kernel methods, other key entities significantly influence the overall efficacy of a quantum kernel. These entities include single-qubit unitary gates, rotational parameters, and entanglement gates. Notably, single-qubit unitary gates, such as phase-shift (U), RX, RY, and RZ gates play a vital role in kernel performance. In this work, we have limited the experiment to a two-qubit, so the entanglement gates remain fixed. Moreover, the impact of different single-qubit rotation gates during the data encoding process is highlighted in the previous studies^21,37^. Therefore, here we only aim to emphasize in detail the role of the rotational factor $(\alpha )$ hyperparameter.

The rotation factor $\alpha$ serves as an intrinsic feature map hyperparameter governing the data encoding process. In this work, fixed values of $\alpha$ are specified for feature maps construction prior to cross-validation, reflecting enhanced quantum kernel design. Importantly, $\alpha$ is not selected based on test-fold performance, ensuring that the evaluation protocol remains unbiased and free from data leakage. Unlike classical SVM hyperparameters, which are tuned via cross-validation, $\alpha$ values are fixed to isolate the representational effects of distinct quantum feature maps.

**Fig. 4:**
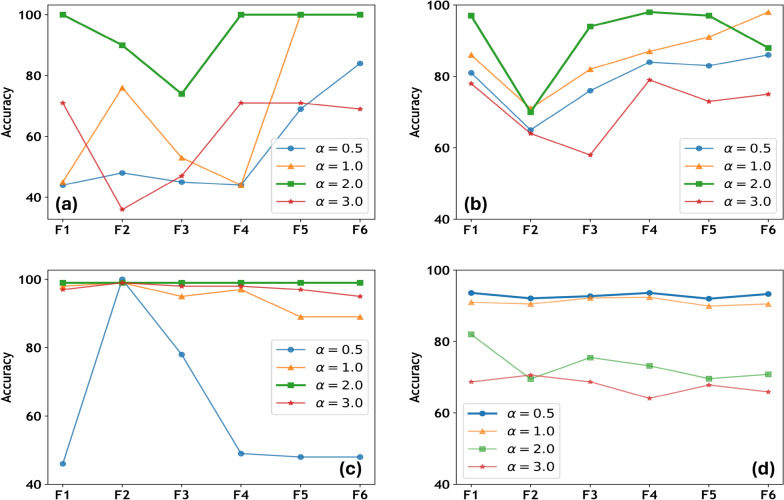
The impact of the rotational factor hyperparameter $(\alpha =0.5,1,2\ and\ 3)$ on test accuracy across the datasets: (**a**) Circle, (**b**) Moon, (**c**) XOR, and (**d**) WBC, evaluated using six different feature maps. Best performances are highlighted with bold lines.

Figure 4 presents a detailed analysis of the role of the $\alpha$-hyperparameter in developing an enhanced quantum model across four different datasets. The illustration reflects the impact of $\alpha$-hyperparameter over quantum kernels (F1–F6) based on the different choices of $\alpha$. The analysis of the $\alpha$-hyperparameter comprises using different set of values 0.5, 1, 2, 3 and are consistently applied across all datasets and kernels to ensure an objective and informative evaluation. Figure 4a presents the hyperparameter analysis for Circle. Circle results indicate that $\alpha =2$ is the most effective choice of the $\alpha$-hyperparameter, consistently outperforming other $\alpha$ values. Specifically, $\alpha =2$ yielded the highest accuracy across feature maps F1 and F4–F6. In contrast, other $\alpha$ values demonstrated notable performance deviations. For example, while $\alpha =1$ performed well with F5 and F6, it showed substantially lower accuracy with F1–F4. Similarly, both $\alpha =0.5$ and $\alpha =3$ found to be the least effective choices for Circle.

**Fig. 5:**
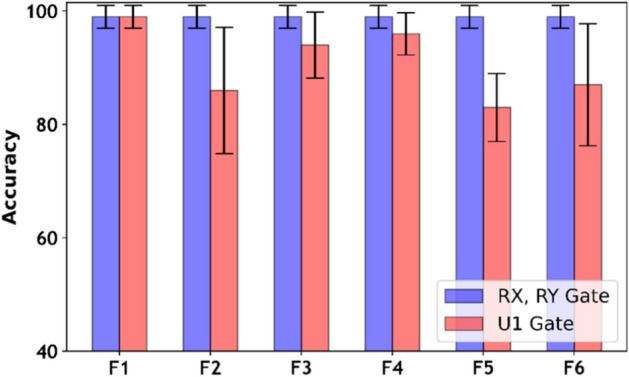
XOR classification accuracy variations using different single qubit rotations for distinct feature maps with $\alpha =2$.

**Fig. 6:**
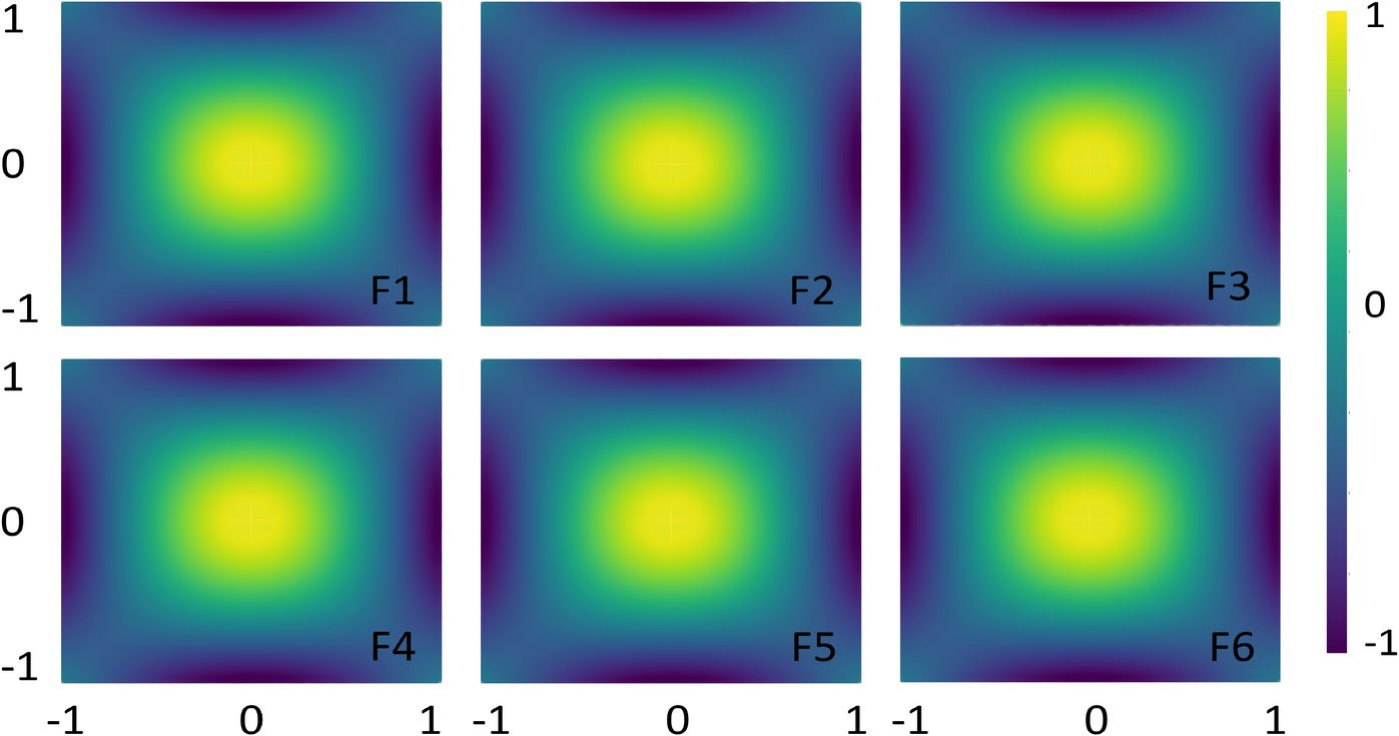
Feature map analysis using six different encoding functions helps predict the data pattern prior to classification performance for Circle.

**Fig. 7:**
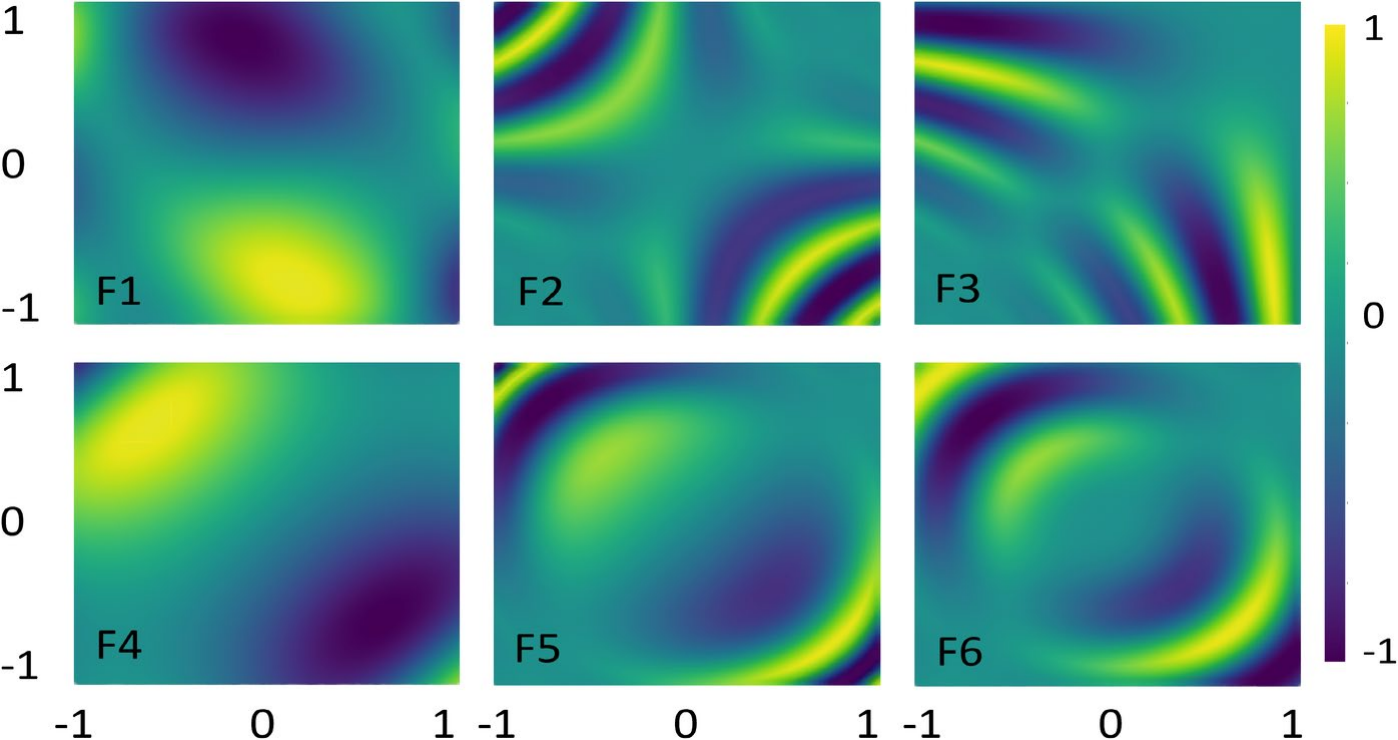
Feature map analysis using six different encoding functions helps predict the data pattern prior to classification performance for Moon.

Figure 4b presents the results for Moon dataset. The plot indicates that $\alpha =2$ yields the most optimal outcomes, with feature maps F1 and F4 achieving the highest accuracy, while F2 and F6 performing the poorest. At $\alpha =1$, only F6 approached the optimal performance, whereas the remaining feature maps were comparatively less effective. In contrast, $\alpha$ values of 0.5 and 3.0 showed the highest deviation from the optimal score, resulting in the weakest performance, and can therefore be considered the least favorable choices for Moon. Figure 4c presents the results for XOR dataset. Similar to Circle and Moon, XOR achieved the optimal performance with $\alpha =2$. Additionally, the plot indicates that the influence of $\alpha$-values is limited, as results across the different feature maps (F1–F6) showed minimal variation. The performance remained consistent across most $\alpha$ values, except for $\alpha =0.5$, which yielded the poorest results.

This could be possible because XOR data distribution can be effectively predicted by the rotational gates, thereby reducing the impact of other entities, such as $\alpha$^37,54^. Furthermore, in Fig. 5, we assessed the rationale through presenting XOR classification variations using different single-qubit rotations with distinct feature maps. The assessment found that rotation along the x-axis (RX gate) and y-axis (RY gate) can predict XOR data patterns better than the rotation along phase shift gate (U1 gate). Moreover, Figure 5 also limits the role of encoding functions along x and y rotations, highlighting the impact of encoding functions with U1 gate. Clearly, the proposed map F1 has again provided significant advantages with U1, RX, and RY gates. Nonetheless, F1 with $\alpha =2$ consistently emerges as the optimal choice for achieving superior results across Circle, Moon, and XOR datasets.

**Fig. 8:**
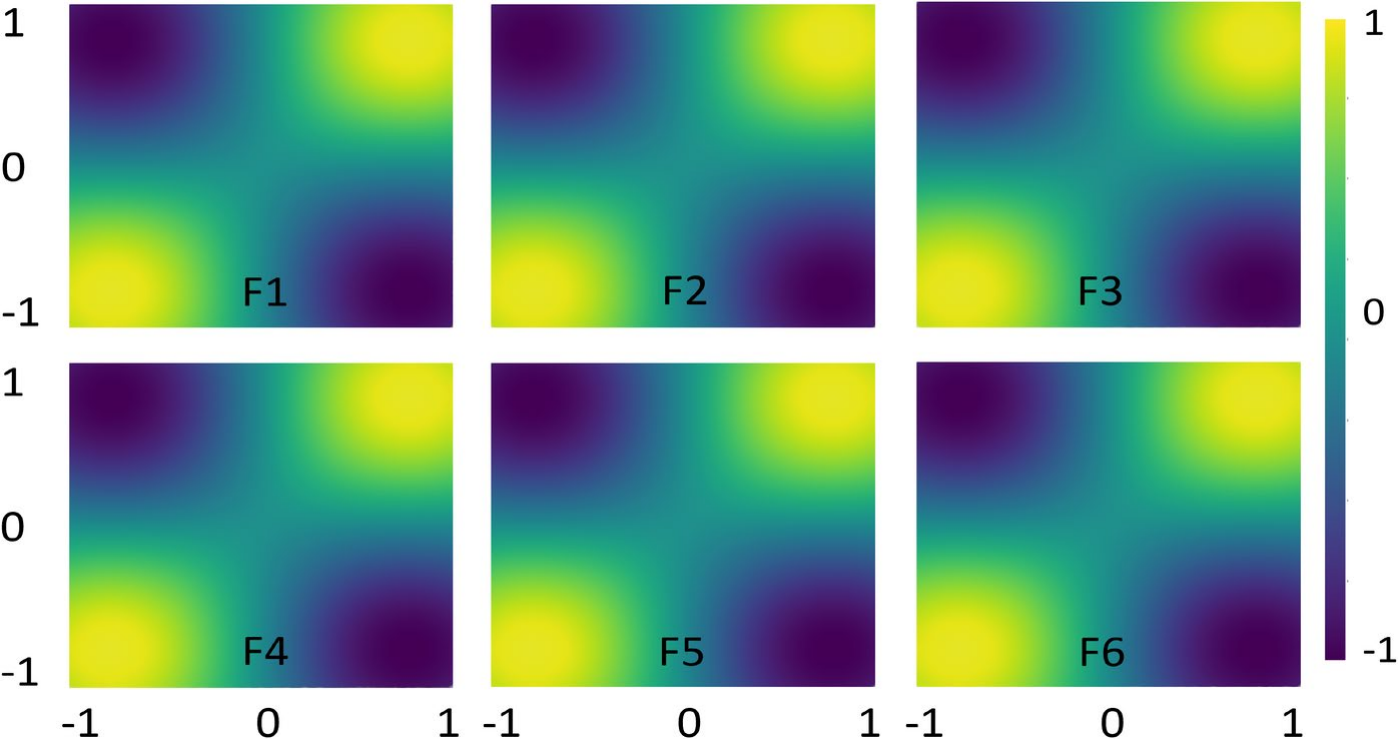
Feature map analysis using six different encoding functions helps predict the data pattern prior to classification performance for XOR.

**Fig. 9:**
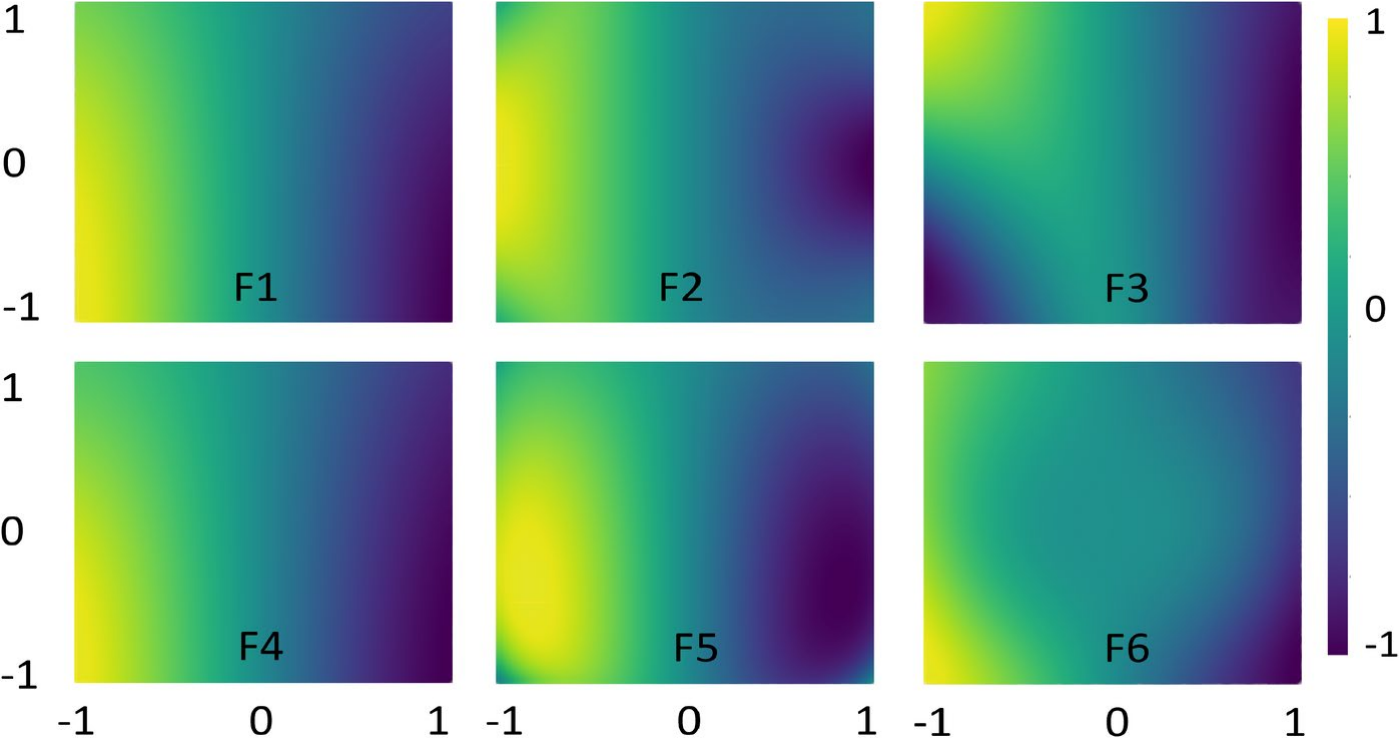
Feature map analysis using six different encoding functions helps predict the data pattern prior to classification performance for WBC.

Figure 4d presents the results for WBC data, which serves as an extension of enhanced quantum kernels design applied to a complex real-world data distribution. Unlike the other datasets analyzed which exhibit either an identifiable data distributions or higher separability, WBC demonstrates neither well-defined data distributions nor clear separability (see data in Fig. 3d). The results in Fig. 4d indicate that the optimal outcome is achieved with $\alpha =0.5$, deviating from the trend of optimality observed with $\alpha =2$ on other datasets. Moreover, the influence of the $\alpha$-hyperparameter appeared more significant, exhibiting higher deviations across WBC outcomes. The choice of $\alpha =1$ showed outcomes closer to the optimal value of $\alpha =0.5$, which followed a similar trend. In contrast, $\alpha =2$ and $\alpha =3$ provided the two inferior choices based on the accuracy obtained. Analyzing WBC highlights the importance of identifying an appropriate hyperparameter, particularly when data distributions lack clear patterns or separability. It is worth highlighting the consistent superior performance of the proposed feature map F1 across the datasets, as well as its ability to outperform other feature maps tuned appropriately. Overall, the hyperparameter setting resulted in enhanced quantum kernel model, leading to better classification outcomes.

Based on the aforementioned analysis, it can be clearly stated that the quantum classifier performance can be improved by enhancing a quantum kernel either through a new data encoding scheme or by identifying suitable hyperparameters^42,48,55^.

### Feature map analysis

This section analyzes feature maps in the feature space by visualizing their role in predicting data distributions. The analysis of the presented feature maps provides initial insights into how feature maps can impact data classification. We employed the optimal set of $\alpha$ values to conduct feature map analyzes using two-dimensional data points following a uniform random distribution. Figure 6 illustrates the impact of each encoding function (feature map) for estimating Circle data patterns. The results indicate that any encoding function can be effectively applied to this data type, as the feature maps successfully capture the data distribution, resulting in improved classification accuracy.

Figure 7 examines the same objective for Moon; however, the encoding functions did not capture Moon distribution as effectively as they did for Circle. This implies that predicting such data patterns is more challenging. Notably, F1 and F4 functions demonstrated closer alignment with the data distribution, which is reflected in the improved classification outcomes discussed earlier in Table 1. In contrast, F2 and F3 exhibited poor performance due to their inferior data prediction. A similar analogy can be drawn for the encoding functions applied to XOR and WBC datasets.

**Fig. 10:**
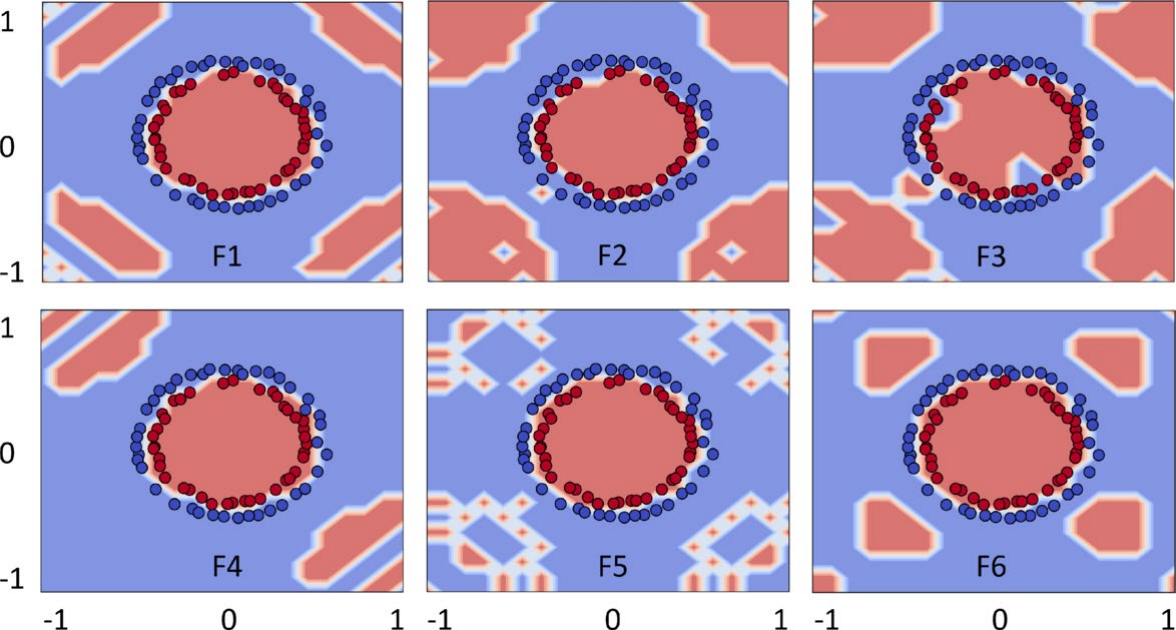
Quantum kernels decision boundary using F1–F6 for Circle. Different feature maps have exhibited variations in decision boundaries, influencing the classification scores.

In Fig. 8, as indicated by the classification metrics, feature maps with tuned hyperparameters effectively predict XOR data distribution with minimal deviations. Figure 9 presents the analysis of encoding maps for WBC dataset, which exhibits a more complex data distribution. F1, F4, and F6 feature maps exhibit similar patterns in representing the data distributions, leading to improved classification scores compared to F2, F3, and F5 which have deviated to the trend. Similar analyzes with $\alpha =2$ are provided in the Supplementary Material (Appendix 4). Finally, the feature map analysis implies that not all feature maps are generally effective for every data distribution. Thus, their suitability must be assessed through hyperparameter tuning, including adjustments to unitary gates, $\alpha$-values, and the structure of the feature map itself.

**Fig. 11:**
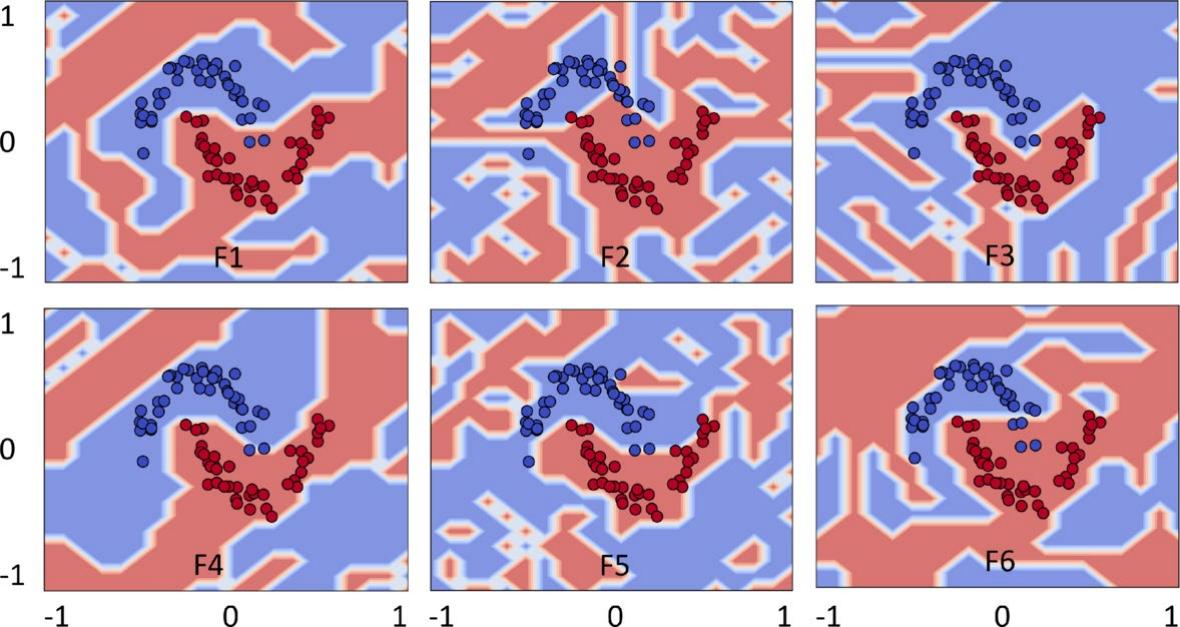
Quantum kernels decision boundary using F1–F6 for Moon. Different feature maps have exhibited variations in decision boundaries, influencing the classification scores.

### Decision boundary

Here we present further visualization about how the role of each feature map played out to discover the useful and complex decision boundary, adding an additional flexibility in quantum kernels. For simplicity, decision boundaries are plotted only for the best possible outcomes of the datasets. Figure 10 demonstrates complex decision boundary formation against each feature map (F1)–(F6) for Circle. Recall that the best solutions are obtained using F1 and F4–F6, and the same evidence is supported by the complex and enhanced decision boundaries formed by quantum kernels. In contrast, the decision boundaries produced by F2 and F3 are slightly distorted, resulting in less accurate quantum kernels.

**Fig. 12:**
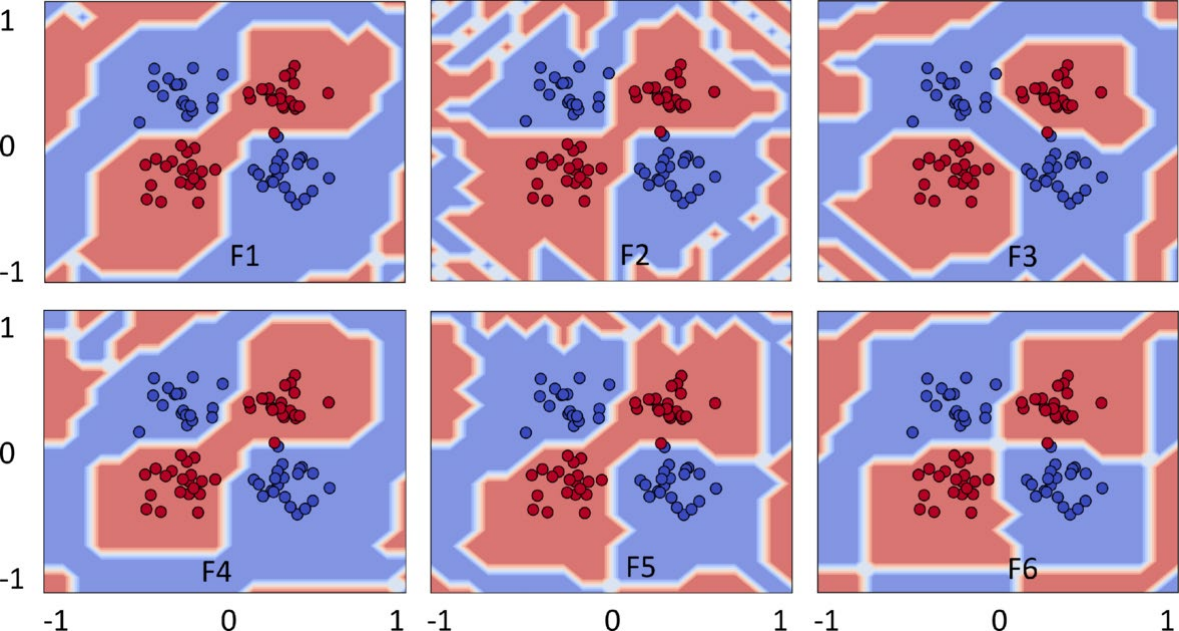
Quantum kernels decision boundary using F1–F6 for XOR. Different feature maps have exhibited variations in decision boundaries, influencing the classification scores.

**Fig. 13:**
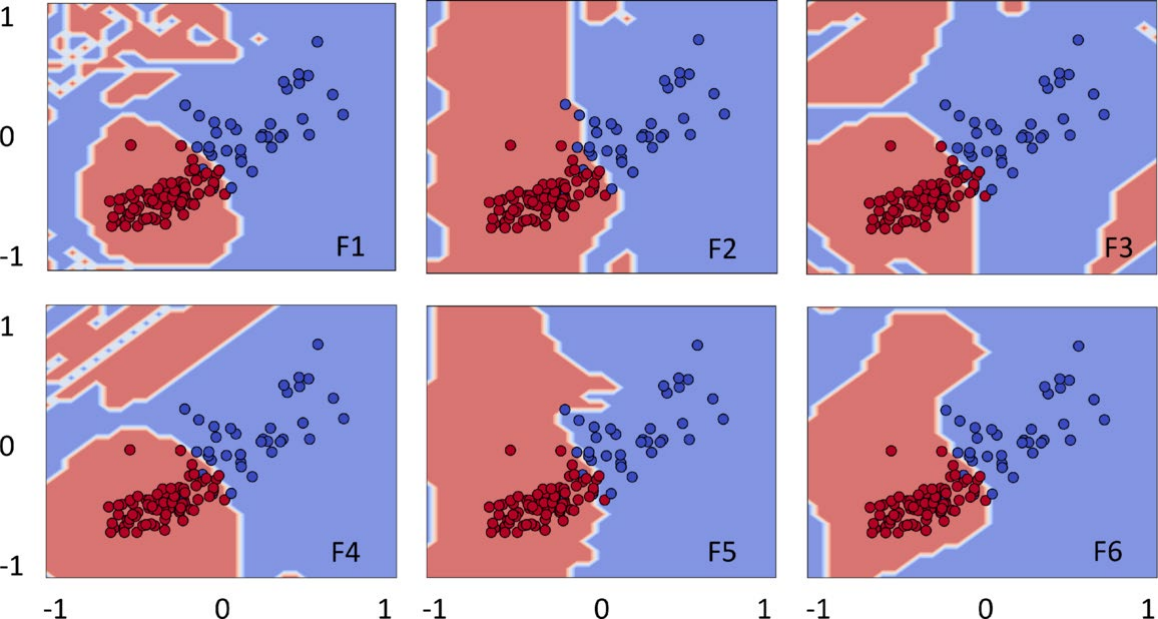
Quantum kernels decision boundary using F1–F6 for WBC. Different feature maps have exhibited variations in decision boundaries, influencing the classification scores.

Figure 11 illustrates the decision boundaries of each feature map (F1–F6) for the Moon. The decision boundaries corresponding to F1 and F4 appear closely aligned with the Moon, supporting the improved model performance. In contrast, the decision boundaries of F2 and F6 appeared poorly aligned, implying the feature maps’ poor performance. Figure 12 shows the decision boundaries corresponding to each feature map (F1)-(F6) for XOR. As anticipated, the decision boundaries generated by the feature maps closely align with the underlying data patterns, thereby supporting effective classification performance. Figure 13 provides complex decision boundary formation against each feature map (F1)–(F6) for WBC. In this case, F1 and F4 have outperformed the other maps, and the same argument is supported by their closely aligned decision boundaries. In contrast, F3 and F5 are the two least performing kernels, and so as their distorted decision boundaries. Similar analyzes with different choices of $\alpha$-value are provided in the Supplementary Material (Appendix 5).

The decision boundary visualizations thus help us present the argument that an appropriate interaction of quantum states through feature maps and appropriate hyperparameter tuning can lead to the formation of a suitable and complex decision boundary. In this study, input ranges and scaling are fixed to ensure robust feature map encoding and to maintain consistency with the formulation used in Suzuki et al.^37^. This setup leads the rotational factor $\alpha$ to effectively act as a primary hyperparameter, determining the sensitivity of a feature map. Therefore, a systematic analysis of how different input ranges and scaling strategies reshape a kernel geometry and the decision boundary would require an extensive future study.

## Conclusion

In this study, we have demonstrated the important steps for constructing enhanced quantum kernels by introducing a new feature map and analyzing existing state-of-the-art feature maps through hyperparameter tuning. The study delves into classification models taken over different datasets which include Circle, Moon, and XOR and further extended to the Wisconsin Breast Cancer (WBC) Diagnostic dataset. The implementation has provided valuable insights for developing enhanced quantum kernels for ML applications. The results are cross-validated and supported by different performance metrics establishing the investigation robustness. The enhanced quantum kernel results are also supported by further visualization using feature map analysis and decision boundary. In summary, results highlight the role of each feature map in approximating various data distributions, implying that different feature maps could be used for different data distributions. Overall, the study presents a detailed analysis of quantum feature maps for building useful quantum models for ML applications.

The study also reflects some important limitations. We investigated the quantum kernel expressivity by analyzing the hyperparameter-tuned feature maps under shallow circuit implementation, and less complex real-world datasets. Furthermore, although the results indicate improved performance, they do not consider the effects of hardware noise, nor do they offer a theoretical perspective on the generalizability of data encoding methods in quantum information theory. Future research incorporating hardware validation is necessary to characterize how quantum noise impacts both the performance and the relative ranking of feature maps.

Our study contributes to the ongoing effort of developing practical quantum computing algorithms for ML problems, although further exploration in this direction remains imperative. Despite significant progress, several challenges remain, such as the development of more robust and unified quantum data encoding schemes and the realization of quantum efficiency. Furthermore, there is a need to assess quantum kernels in terms of their global advantages, particularly in addressing scalability and providing speed-ups for large-scale implementations. Future research should focus on the optimization of quantum kernels and sensitivity analysis on a larger scale, particularly using real-world datasets to address practically relevant problems. Additionally, exploring quantum-inspired algorithms with more generalized qubits is essential for advancing quantum scalability as well as for quantum readiness.

## Supplementary Information

Supplementary Information.

## Data Availability

The Breast Cancer Wisconsin (Diagnostic) data is available in Kaggle as well as can be obtained from the UCI Machine Learning Repository: Breast Cancer Wisconsin (Diagnostic)^56^.
