# Supplementary material for: Comparative performance analysis of quantum feature maps for quantum kernel-based machine learning
Source: Sci Rep. 2026 Feb 10;16:8142. doi: 10.1038/s41598-026-39392-9 (PMC12960865; doi:10.1038/s41598-026-39392-9)

Comparative Performance Analysis of Quantum Feature Maps for Quantum Kernel-based Machine Learning

Appendix 1

Algorithm 1: Quantum Kernel Algorithm

---

1. **Input:** Dataset $\{\overrightarrow{x_i}, y_i\}_{i=1}^{N}$
2. Define a two-qubit quantum feature map $\mathcal{U}_{\Phi(\vec{x})}$ with encoding functions $\Phi(\vec{x})$ and α-hyperparameter
3. **For** each pair of data points $(\vec{x}_i, \vec{x}_j)$, do:

   3.1 Construct the overlap circuit using the quantum feature map implementation

   3.2 Apply $\mathcal{U}_{\Phi(\vec{x}_i)}$ followed by the $\mathcal{U}^{\dagger}_{\Phi(\vec{x}_j)}$

   3.3 Estimate kernel entry $\boldsymbol{K}_{\mathrm{ij}} = \left| \left\langle 0 \right| \otimes^2 \mathcal{U}^{\dagger}_{\Phi(\vec{x}_j)} \, \mathcal{U}_{\Phi(\vec{x}_i)} \left| 0 \right\rangle \otimes^2 \right|^2$

   via projective measurement
4. **End** for
5. Construct the quantum kernel matrix $\boldsymbol{K}$
6. Train SVM using $\boldsymbol{K}$ as a precomputed kernel with the scikit-learn SVC implementation.
7. **Output:** Trained quantum kernel classifier

Appendix 2

A complete illustration of a quantum feature map circuit design implementation on the PennyLane simulator, highlighting the number of qubits, layers, and entanglement sequence for quantum kernel estimation, is provided.

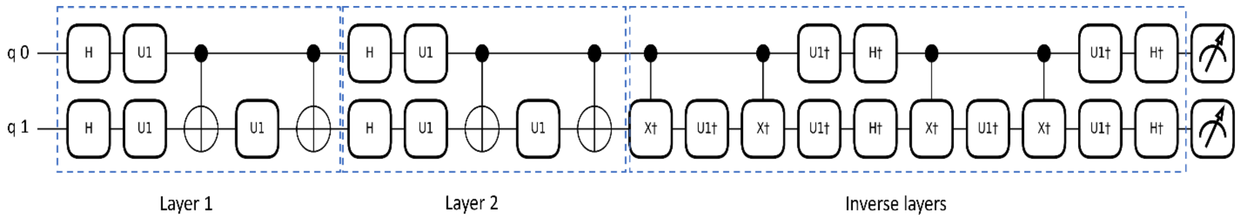

Appendix 3

Different hyperparameters value optimized using grid search cross-validation approach in Table 1. Hyperparameter tuning was conducted using a grid search cross-validation approach to determine the optimal values for various SVM kernels and ML classifiers.

Data	Model	Hyperparameter(s)	Estimates/Values
Circle	Linear (Lin)	Regularization parameter (C)	0.1
	Radial Basis Function (RBF)	Regularization parameter (C), and Kernel coefficient (γ)	2, 1
	Decision Tree (DT)	Depth (tree depth)	8
	Random Forest (RF)	Depth (tree depth), Estimator (trees in the forest)	8, 13
	AdaBoost (AB)	Depth (base estimator), Estimator	4, 14
	MLP	Hidden nodes, Iterations, Activation function, Solver	10, 500, ReLU, Adam
Moon	Linear (Lin)	Regularization parameter (C)	2
	Radial Basis Function (RBF)	Regularization parameter (C), and Kernel coefficient (γ)	5, 1
	Decision Tree (DT)	Depth (tree depth)	6
	Random Forest (RF)	Depth (tree depth), Estimator (trees in the forest)	9, 14
	AdaBoost (AB)	Depth (base estimator), Estimator	3, 5
	MLP	Hidden nodes, Iterations, Activation function, Solver	10, 500, ReLU, Adam
XOR	Linear (Lin)	Regularization parameter (C)	0.5
	Radial Basis Function (RBF)	Regularization parameter (C), and Kernel coefficient (γ)	0.5, 0.5
	Decision Tree (DT)	Depth (tree depth)	7
	Random Forest (RF)	Depth (tree depth), Estimator (trees in the forest)	11, 20
	AdaBoost (AB)	Depth (base estimator), Estimator	2, 9
	MLP	Hidden nodes, Iterations, Activation function, Solver	10, 500, ReLU, Adam
WBC	Linear (Lin)	Regularization parameter (C)	3
	Radial Basis Function (RBF)	Regularization parameter (C), and Kernel coefficient (γ)	2, 0.01
	Decision Tree (DT)	Depth (tree depth)	4
	Random Forest (RF)	Depth (tree depth), Estimator (trees in the forest)	1, 8
	AdaBoost (AB)	Depth (base estimator), Estimator	3, 3
	MLP	Hidden nodes, Iterations, Activation function, Solver	10, 500, ReLU, Adam

Appendix 4

Feature map analysis with $\alpha = 2.0$ using six different encoding functions is helpful in investigating data patterns prior to the classification for the Wisconsin Diagnostic Breast Cancer (WBC) dataset. The distinct maps suggest different data patterns, except F1 which appeared to be a slightly closer to WBC data, F2-F6 maps appeared hard to adapt the distributions. Therefore, F2-F6 provided weak quantum kernels with other values of α-hyperparameter.

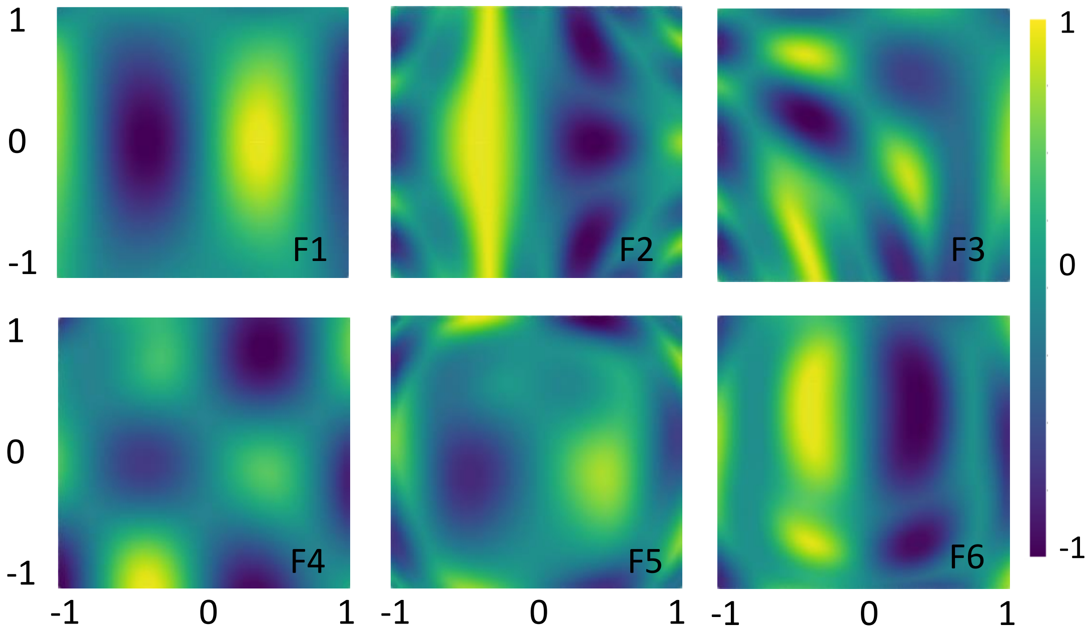

Appendix 5

Quantum kernels decision boundary using F1-F6 for WBC with $\alpha = 2.0$. Different feature maps have exhibited variations in decision boundaries, influencing the classification scores. The plot suggests the distorted decision boundary, making them unsuitable for beneficial classifiers with $\alpha = 2.0$.

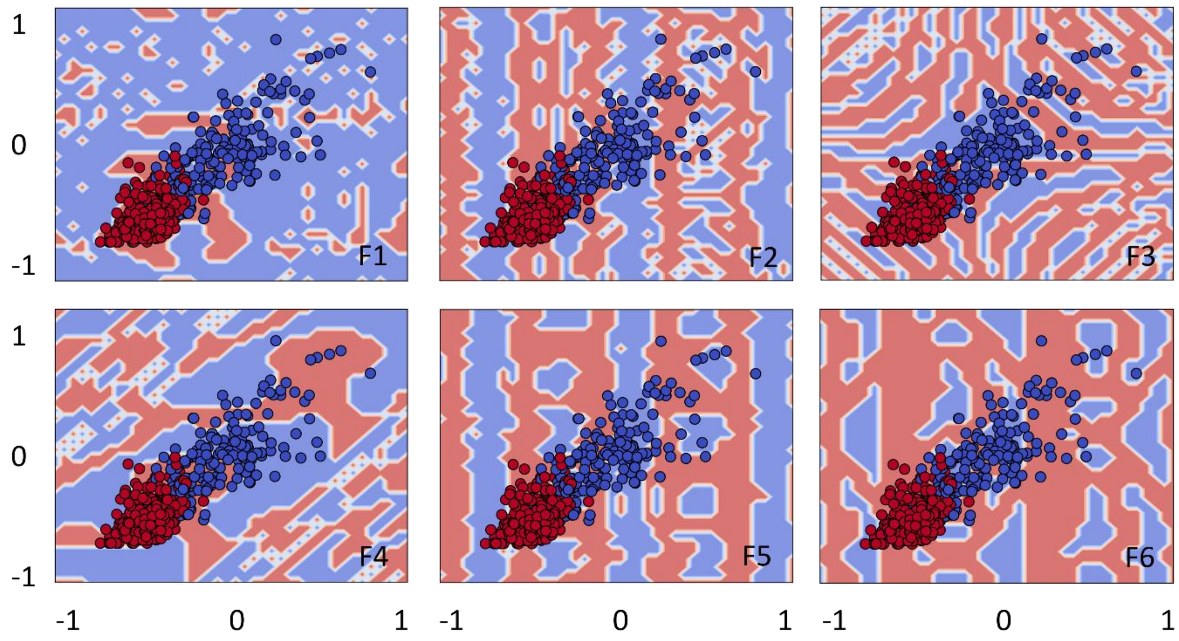

Supplement: Supplementary file 1 — Supplementary Information. [file 41598_2026_39392_MOESM1_ESM.pdf]
